# Global Investigation of TBL Gene Family in Rose (*Rosa chinensis*) Unveils *RcTBL16* Is a Susceptibility Gene in Gray Mold Resistance

**DOI:** 10.3389/fpls.2021.738880

**Published:** 2021-10-01

**Authors:** Yu Tian, Shiya Zhang, Xintong Liu, Zhao Zhang

**Affiliations:** Beijing Key Laboratory of Development and Quality Control of Ornamental Crops, Department of Ornamental Horticulture, China Agricultural University, Beijing, China

**Keywords:** *Rosa* sp., TBL, *Botrytis cinerea*, gene family, expression, *S*-gene

## Abstract

The TRICHOME BIREFRINGENCE-LIKE (TBL) family is an important gene family engaged in the *O*-acetylation of cell wall polysaccharides. There have been a few reports showing that TBL participated in the resistance against phytopathogens in Arabidopsis and rice. However, no relevant studies in rose (*Rosa* sp.) have been published. In this study, a genome-wide analysis of the *TBL* gene family in rose was presented, including their phylogenetic relationships, gene structure, chromosomal positioning, and collinearity analysis. The phylogenetic analysis revealed a total of 50 *RcTBL* genes in the rose genome, and they are unevenly distributed across all seven chromosomes. The occurrence of gene duplication events suggests that both the whole genome duplication and partial duplication may play a role in gene duplication of *RcTBL*s. The analysis of Ka/Ks showed that the replicated *RcTBL* genes underwent mainly purifying selection with limited functional differentiation. Gene expression analysis indicated that 12 *RcTBL*s were down-regulated upon the infection of *Botrytis cinerea*, the causal agent of the gray mold disease of rose. These *RcTBL*s may be a sort of candidate genes for regulating the response of rose to *B. cinerea*. Through virus-induced gene silencing, *RcTBL16* was shown to be associated with susceptibility to gray mold in rose. Through this study, meaningful information for further studies on the function of the TBL protein family in rose is provided.

## Introduction

The cell wall is particularly important in plant growth and development because it maintains the form of the plant cell, allows intercellular communication, responds to external environmental variables, and interacts with pathogenic microorganisms (Keegstra, 2010; Xin et al., 2010). The plant cell wall has a complicated and dynamic structure, which is mainly composed of polysaccharide polymer, protein and lignin. *O*-acetylation occurs extensively in the plant cell wall, most notably with hemicelluloses, pectins, and lignins. The replacements of *O*-acetyl group usually happen on various specific glycosyl residues of cell wall polysaccharides. In addition, cell wall polysaccharides can be either mono- or diacetylated, and the extent of *O*-acetylation depends on species, tissue type, and growth status of plants.

The biosynthetic pathway and function of *O*-acetylation of cell wall polysaccharides have not yet been fully understood. Modifications in *O*-acetylation levels are known to alter plant growth and development, as well as their defense against pathogens, and that this effect is most likely achieved by altering the cell wall structure. The REDUCED WALL ACETYLATION (RWA) protein, as well as the ALTERED XYLOGLUCAN9 protein and TRICHOME BIREFRINGENCE-LIKE (TBL) protein families, has been identified as being involved in the *O*-acetylation pathway of plant cell walls. RWA proteins could be acetyl donor transporters, transporting acetyl CoA into the Golgi apparatus (Manabe et al., 2011, 2013). AXY9 protein may be required for *O*-acetylation of cell wall polysaccharides by producing acetylation intermediates (Schultink et al., 2015).

Unlike the RWA and AXY9 proteins, a number of TBLs have been identified as polysaccharide acetyltransferases, catalyzing the *O*-acetylation of specific cell wall polymers including xylan (Urbanowicz et al., 2015; Zhong et al., 2017a; Zhong et al., 2018a,d), xyloglucan (Zhong et al., 2018c,e; Zhong et al., 2020), mannan (Zhong et al., 2018b), and pectin (Vogel et al., 2004b; Bischoff et al., 2010a; Stranne et al., 2018a; Chiniquy et al., 2019a). TBL proteins have conserved Asp-x-x-His (DxxH) motif and Gly-Asp-Ser (GDS) motif that is required for acetyltransferase activity (Bischoff et al., 2010b), since a mutation in either the GDS or DXXH motif could cause TBL proteins to lose their function completely (Zhong et al., 2017a; Zhong et al., 2018e). Studies of *tbl* mutants in Arabidopsis have demonstrated that dwarfism, stem weakness, and stunted growth of plants are associated with the lack of the TBL genes (Bischoff et al., 2010a; Xiong et al., 2013; Schultink et al., 2015), implying that TBL is critical for plant development. Besides, TBL proteins are related to abiotic stress in plants. Compared with wild-type Arabidopsis plant, the cold tolerance of *esk1* increased significantly (Xin et al., 2010), while *tbl10* showed enhanced drought resistance (Stranne et al., 2018a). Furthermore, TBL proteins have also been associated with plant defense against pathogens. The reduction of *O*-acetyl degree of *pmr5* in Arabidopsis may lead to its enhanced resistance to powdery mildew (Vogel et al., 2004b; Chiniquy et al., 2019a). According to a recent research, simultaneous mutation of the *OsTBL1* and *OsTBL2* genes in rice leads to lower acetylation levels and higher vulnerability to leaf blight disease (Gao et al., 2017).

Rose (*Rosa* sp.) is the most important cut flower in the world, with 8,500 hectares of cut-flower rose cultivation worldwide, with an annual production of over 15 billion stems (Bendahmane et al., 2013), and sales of more than $11 billion (Zlesak, 2007). Gray mold caused by *Botrytis cinerea* is the most devastating disease mainly infecting the flower of rose and affects the production of cut rose all over the world (Gleason and Helland, 2003). The cell wall is the initial barrier that pathogens meet when penetrating the plant, and alterations in cell wall structure might affect the plant’s defense against these microbes. *O*-acetylation is one of the most important modifications of cell wall polymer. TBL proteins, as the main gene family involved in cell wall *O*-acetylation, may influence the resistance or susceptibility of plants to pathogen by varying the degree of cell wall acetylation. However, no research on the function of the TBL gene at the plant genome-wide level has been conducted thus far. We performed the first genome-wide analysis of the *RcTBL* family in rose in this study. Furthermore, a virus-induced gene silencing (VIGS) has confirmed that *RcTBL16* was involved in *B. cinerea* susceptibility.

## Materials and Methods

### Characterization of Putative TBL Proteins in Rose

We downloaded the complete rose genome sequence and CDS sequence from the website https://lipm-browsers.toulouse.inra.fr/pub/RchiOBHm-V2/ to construct a local genome database. In order to identify non-redundant *RcTBL* genes in rose genome, the HMM profile of the PC-Esterase domain was obtained from Pfam (PF13839^1^). Then, using this HMM profile as a query, by searching the rose genome, all sequences were confirmed to contain a PC-Esterase domain with an E value of <1e^-3^ in rose. The distribution of all *RcTBL* genes on chromosomes was mapped by mapchart 2.2 software.

### Gene Structure and Phylogenetic Analyses

The gene structure map of *RcTBL* was completed using TBtools (Chen et al., 2018) by means of the rose genome annotation file and protein sequences. Multiple comparisons of *RcTBL* amino acids (aa) were performed using the ClustalW default parameters. A phylogenetic analysis of *RcTBL*s then was carried out in MEGA-6.0 software by the maximum likelihood (ML) method, with 1,000 bootstrap replications, JTT with Freqs (+F) model and 50% partial deletion. Other parameters were set by default. MEGA 6.0 was also applied to construct the unrooted ML trees of TBL proteins from Arabidopsis and rose, the parameter settings being consistent with the separate phylogenetic analysis of *RcTBL*s.

### Collinearity Analyses

For the purpose of identifying the collinearity of *RcTBL*s, the genome sequence of rose was downloaded on a local server, and then we used a Multiple Collinearity Scan toolkit (Wang et al., 2012) to determine the microsyntenic relationships between *RcTBL* genes. Furthermore, collinearity scanning (e-value of <1e^−10^) was used to evaluate the microsynteny relationships.

### Calculation of Ratios of Nonsynonymous (Ka) to Synonymous (Ks) Nucleotide Substitutions

We used TBtools (Chen et al., 2018) to calculate Ks and Ka nucleotide substitution rates. The Ka/Ks ratio of duplicated gene pairs was calculated to determine the selection pattern driving the evolution of *RcTBL*.

### Expression of *RcTBL*s in Response to *B. cinerea*

RNA-Seq data from rose petals under *B. cinerea* infection were obtained from the National Center for Biotechnology Information database as accession number PRJNA414570 (Liu et al., 2018). The materials for RNA-seq are rose petal discs infected with *B. cinerea* at 30h post inoculation (hpi) and 48hpi, with three biological repeats for both infected and control treatments at each time point. Clean sequencing reads were mapped to the *Rosa chinensis* ‘Old Blush’ reference genome.^2^ We calculated the gene expression levels of *RcTBL*s by Reads per kb per million reads and performed a Log2 fold change-based differentially expressed gene analysis by DEseq2.

For validating the RNA-Seq outcomes, RT-qPCR was used to analyze the expression of five *RcTBL* genes. Total RNA was extracted from rose petals at 30hpi and 48hpi, respectively. As described previously (Wu et al., 2016), the hot borate method was used to extract total RNA. Then, first-strand cDNA was synthesized using HiScript II Q Select RT SuperMix (Vazyme) in a 20-μl reaction volume with 1μg of DNase-treated RNA. RT-qPCR reaction was run using SYBR Green Master Mix (Takara) and detection was achieved in a StepOnePlus real-time PCR system (Thermo Fisher Scientific). We used *RcUBI2* as an internal control and conducted expression analysis using the delta–delta-Ct method of calculation. All primers used as RT-qPCR are listed in Supplementary Table S1.

### VIGS and *B. cinerea* Inoculation Assays

In order to obtain the *pTRV2-RcTBL* constructs, a~200bp fragment from the coding region of *RcTBL*s was amplified with specific primer pairs and subsequently cloned into the *pTRV2* vector (Liu et al., 2002). VIGS was established as previously described (Cao et al., 2019). Briefly, separated petals were obtained from the outermost whorls of cut roses in the second stage of flowering. A 15mm disc was then punched from the center of each petal. *Agrobacterium tumefaciens* consisting of *pTRV1* and *pTRV2* constructs were mixed in a 1:1 ratio and vacuumed infiltrated into petal discs. Petals were inoculated with *B. cinerea* on day 6 after TRV infection and photographed 60h later to obtain images with disease lesions, which were statistically analyzed by ImageJ. Each gene was silenced at least three times with 48 petals as a replicate. Student’s *t* test was performed to determine the significance of lesion size. After photographing, the petal samples were collected for further validating of silencing efficiency by RT-qPCR. The primers used to detect silencing efficiency of *RcTBL16* or *RcTBL35* are the same primers as those used to detect expression in response to *B. cinerea*, and listed in Supplementary Table S1.

## Results

### Identification of *RcTBL* Genes in Rose

As previously stated, the TBL protein family is distinguished by a conserved GDS signature and DXXH motif, as well as an N-terminal transmembrane domain in most of the cases (Bischoff et al., 2010a,b). A total of 61 candidate RcTBL proteins were obtained in rose by the Hidden Markov model (HMM) profile of PC-Esterase domain (PF13839) contained two conservative motifs of the TBL protein family. All candidate sequences less than 150 amino acids and without the complete conserved motifs were removed; finally, we obtained a total of 50 RcTBLs.

All *RcTBL*s can be mapped to rose chromosomes; we designated the genes *RcTBL01* to *RcTBL50* according to their chromosome order. The protein length of RcTBLs varies greatly. Of the 50 RcTBLs, RcTBL23 is the longest protein with 630 aa, while the shortest is RcTBL15 with 154 aa. The average length of proteins in RcTBL family is 409 aa. Details of the *RcTBL* genes, with gene number, chromosomal location, introns, exons, CDS and aa length are listed in Table 1.

**Table 1:** Members of the *RcTBL* gene family as predicted in *Rosa chinensis* genome sequence.

Gene	Accession number^1^	Chr^2^	Position^3^	Intron	Exon	CDS(bp)	Amino acids
RcTBL01	RchiOBHm_Chr1g0332411	1	23.56	4	5	1,152	383
RcTBL02	RchiOBHm_Chr1g0359011	1	50.99	2	3	1,368	455
RcTBL03	RchiOBHm_Chr1g0370451	1	59.94	4	5	1,482	493
RcTBL04	RchiOBHm_Chr1g0371061	1	60.41	5	6	1,302	433
RcTBL05	RchiOBHm_Chr1g0375151	1	62.68	3	3	1788	595
RcTBL06	RchiOBHm_Chr2g0095401	2	8.34	5	5	1,599	532
RcTBL07	RchiOBHm_Chr2g0120351	2	33.07	4	4	1,365	454
RcTBL08	RchiOBHm_Chr2g0121951	2	34.79	5	4	1,509	502
RcTBL09	RchiOBHm_Chr2g0129071	2	44.73	4	5	1,122	373
RcTBL10	RchiOBHm_Chr2g0131401	2	47.54	4	5	1,056	351
RcTBL11	RchiOBHm_Chr2g0131411	2	47.57	3	4	768	255
RcTBL12	RchiOBHm_Chr2g0131441	2	47.65	4	5	1,092	363
RcTBL13	RchiOBHm_Chr2g0131461	2	47.69	3	4	624	207
RcTBL14	RchiOBHm_Chr2g0150421	2	68.08	4	5	1,146	381
RcTBL15	RchiOBHm_Chr2g0163731	2	79.08	0	1	465	154
RcTBL16	RchiOBHm_Chr2g0163771	2	79.09	1	2	1,275	424
RcTBL17	RchiOBHm_Chr2g0163781	2	79.10	1	2	1,350	449
RcTBL18	RchiOBHm_Chr2g0170621	2	84.51	2	3	1,278	425
RcTBL19	RchiOBHm_Chr3g0461751	3	9.73	5	6	1,332	443
RcTBL20	RchiOBHm_Chr3g0461761	3	9.74	4	5	1,425	474
RcTBL21	RchiOBHm_Chr3g0462741	3	10.34	5	6	1,308	435
RcTBL22	RchiOBHm_Chr3g0474891	3	20.68	2	3	792	263
RcTBL23	RchiOBHm_Chr3g0479331	3	25.17	4	5	1893	630
RcTBL24	RchiOBHm_Chr4g0398501	4	15.04	4	5	1,311	436
RcTBL25	RchiOBHm_Chr4g0398521	4	15.07	4	5	1,533	510
RcTBL26	RchiOBHm_Chr4g0417821	4	43.12	3	4	1,365	454
RcTBL27	RchiOBHm_Chr4g0424421	4	50.04	0	1	1,056	351
RcTBL28	RchiOBHm_Chr4g0433581	4	57.66	2	3	555	184
RcTBL29	RchiOBHm_Chr4g0442021	4	63.74	4	5	1863	620
RcTBL30	RchiOBHm_Chr4g0444361	4	65.13	1	2	1,353	450
RcTBL31	RchiOBHm_Chr5g0010331	5	6.84	4	5	1,113	370
RcTBL32	RchiOBHm_Chr5g0025161	5	19.14	4	3	549	182
RcTBL33	RchiOBHm_Chr5g0029581	5	23.19	4	5	1,137	378
RcTBL34	RchiOBHm_Chr5g0029591	5	23.20	4	5	1,023	340
RcTBL35	RchiOBHm_Chr5g0029601	5	23.20	4	5	1,143	380
RcTBL36	RchiOBHm_Chr5g0029611	5	23.21	4	5	1,086	361
RcTBL37	RchiOBHm_Chr5g0031471	5	25.06	4	5	1,251	416
RcTBL38	RchiOBHm_Chr5g0070191	5	76.02	4	5	1,161	386
RcTBL39	RchiOBHm_Chr5g0081011	5	87.06	2	3	549	182
RcTBL40	RchiOBHm_Chr5g0081071	5	87.07	5	6	1,638	545
RcTBL41	RchiOBHm_Chr5g0081121	5	87.16	4	5	1,578	525
RcTBL42	RchiOBHm_Chr5g0081131	5	87.16	4	5	1761	586
RcTBL43	RchiOBHm_Chr6g0247241	6	3.06	5	6	1,260	419
RcTBL44	RchiOBHm_Chr6g0247271	6	3.12	6	7	1,317	438
RcTBL45	RchiOBHm_Chr6g0269281	6	26.54	3	4	1,194	397
RcTBL46	RchiOBHm_Chr6g0277131	6	39.52	3	3	1,323	440
RcTBL47	RchiOBHm_Chr6g0291221	6	54.42	3	4	1,302	433
RcTBL48	RchiOBHm_Chr7g0195391	7	13.24	2	3	1,332	443
RcTBL49	RchiOBHm_Chr7g0217351	7	35.23	3	4	756	251
RcTBL50	RchiOBHm_Chr7g0241111	7	67.11	4	5	1,542	513

1 *Available at:* *https://lipm-browsers.toulouse.inra.fr/pub/RchiOBHm-V2/*

2 *Chromosome*.

3 *Starting position (Mb)*.

### Chromosomal Locations, Whole-Genome Duplication, and Microsynteny

**Figure 1:**
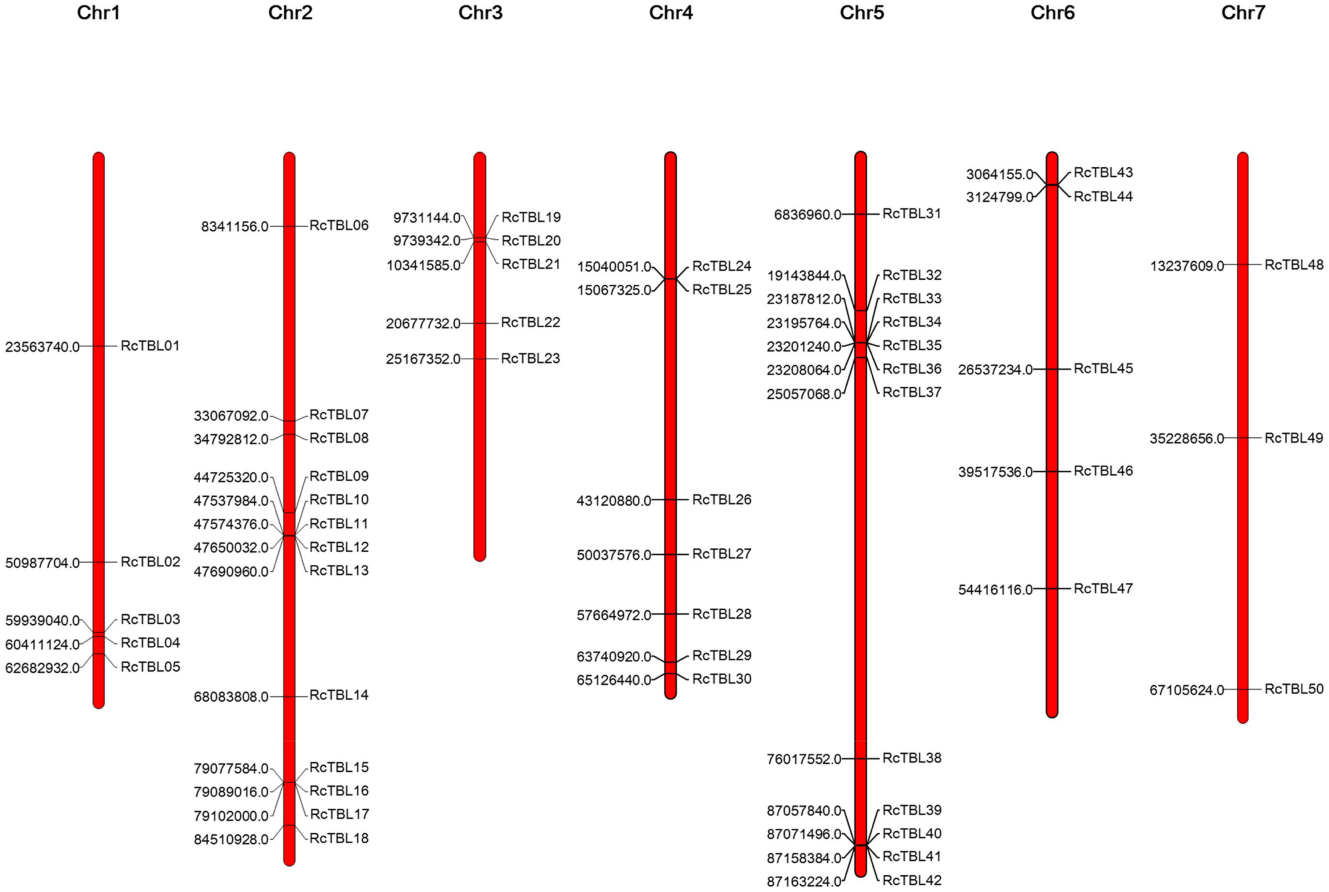
Chromosome localization of rose TBL family members. The distribution of *RcTBL*s is shown on the seven chromosomes of *Rose chinensis*. The starting position (bp) of each *RcTBL* is listed on the left side of the chromosomes.

**Figure 2:**
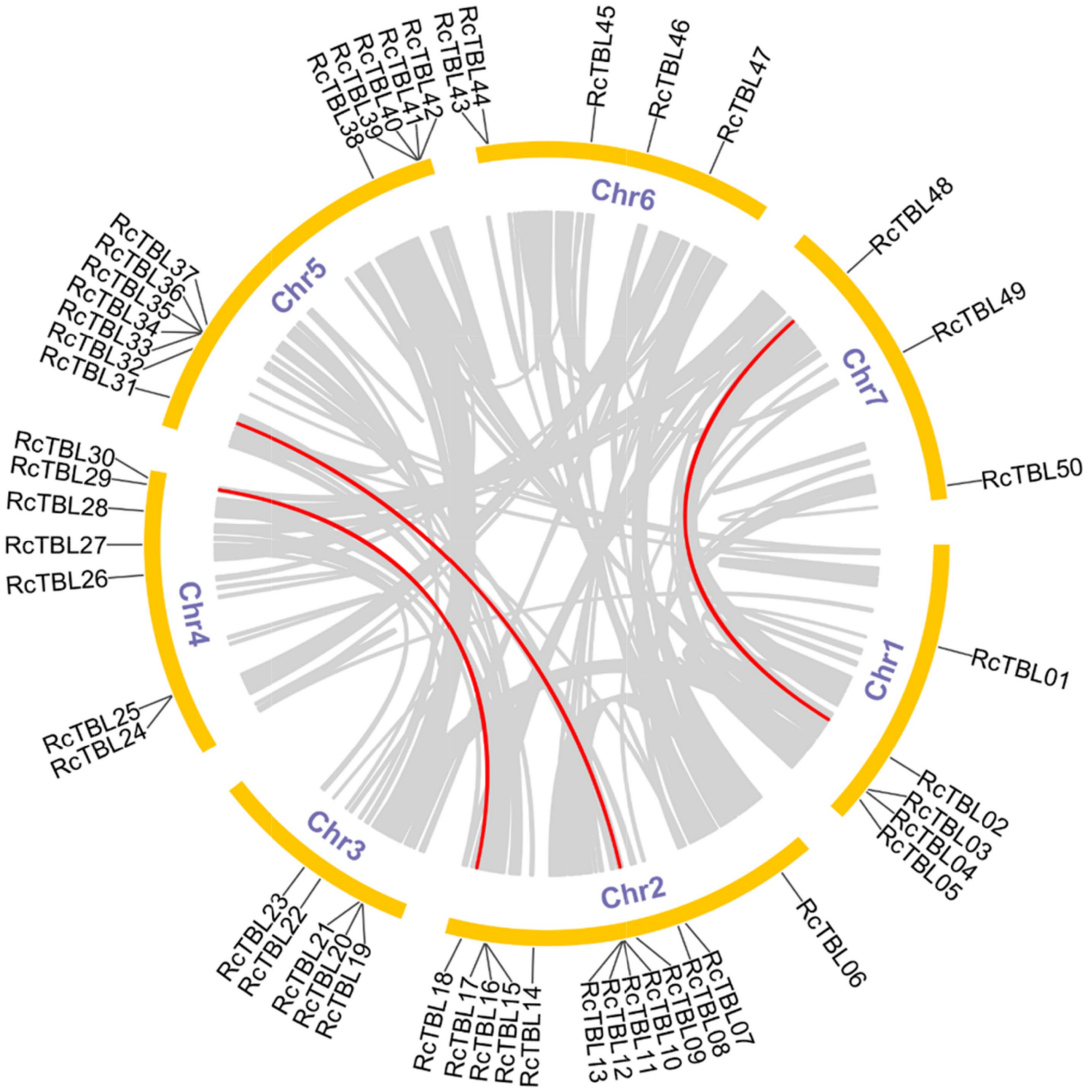
Microsyntenic analyses of the rose TBL protein family members in the *R. chinensis* genome. Circular visualization of rose TBL protein family members is mapped onto different chromosomes by using Circos. The red lines indicate rose *TBL* genes with a syntenic relationship. The gray lines represent all syntenic blocks in the genome of *R. chinensis*.

All 50 *RcTBL* genes were distributed unevenly across seven rose chromosomes (Figure 1), with chromosome 2 having the highest density, gathering 13 *RcTBL* genes, followed by chromosome 5 with 12 *RcTBL* genes clustered on it, and chromosome 7 having the fewest *RcTBL* genes with only three members. We further investigated the duplication events in *RcTBL*s. Tandemly duplicated genes were defined as arrays of two or more homologous genes in the 100kb range. Three *RcTBL* gene pairs were discovered in the rose genome, each on a different chromosome, suggesting that segmental duplication occurs within these regions in rose. Collinearity analyses of the *RcTBL* genes on the chromosome are depicted in Figure 2.

To explore the selective constraints among duplicated *RcTBL* genes, the ratio of nonsynonymous (Ka) to synonymous (Ks) nucleotide substitutions (Ka/Ks ratio) of three pairs of duplicated genes (Table 2) was calculated. Typically, Ka/Ks >1 is consistent with positive selection, while Ka/Ks <1 indicates purifying selection. Ka/Ks <1 for all three duplicated gene pairs (Table 2) suggested that the primary driver of gene evolution in the *RcTBL* family was purifying selection.

**Table 2:** Duplication analysis of the *RcTBL* gene family.

Gene 1	Gene 2	Ka	Ks	Ka_Ks	Effective Len	Average S-sites	Average N-sites
RcTBL18	RcTBL30	0.645328	NaN	NaN	1,257	275.3333333	981.6666667
RcTBL09	RcTBL31	0.327007	2.080681	0.157164	1,107	252.4166667	854.5833333
RcTBL02	RcTBL48	0.317547	1.749673	0.181489	1,299	283.75	1015.25

### Phylogenetic and Gene Structural Analysis of Rose TBL Genes

**Figure 3:**
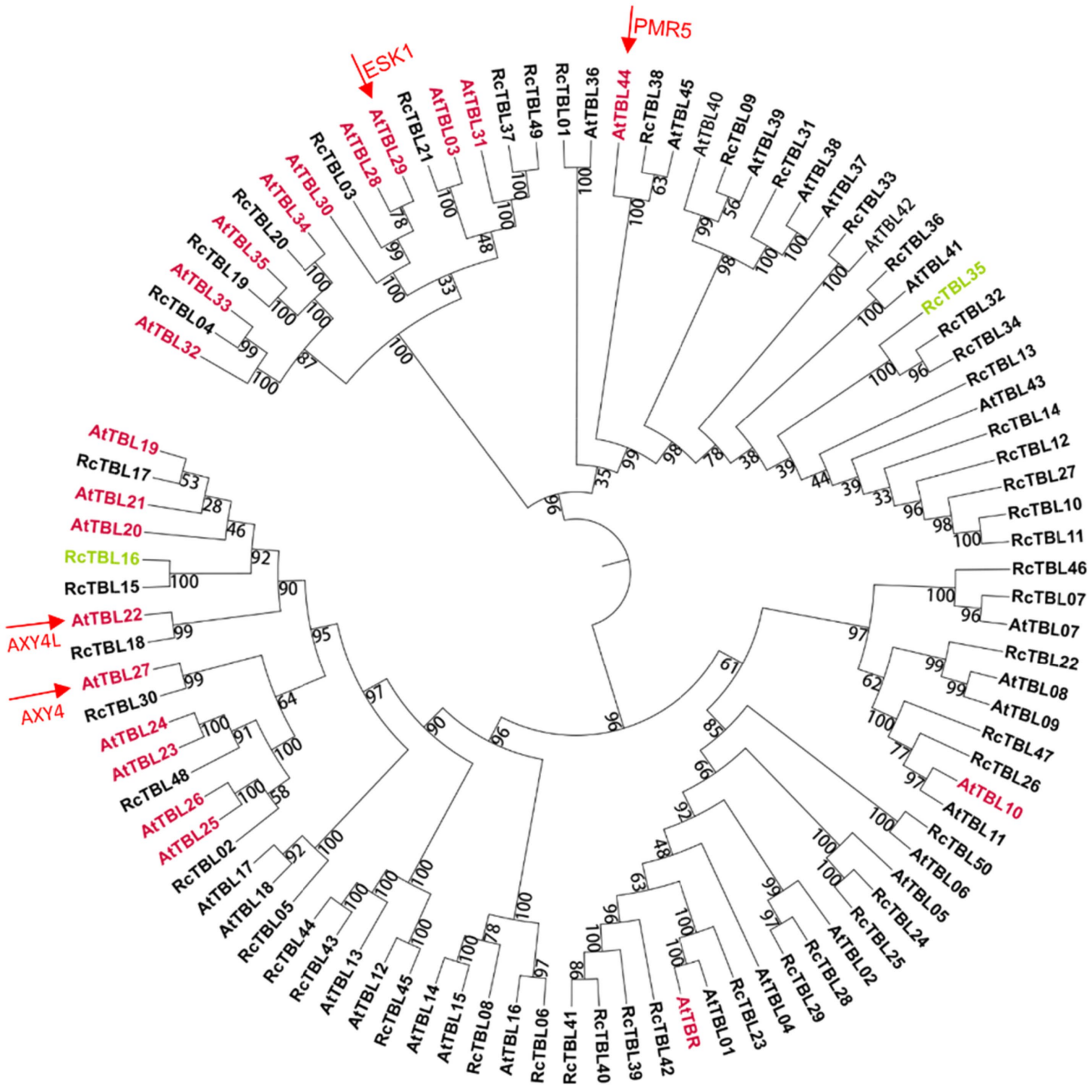
Phylogenetic analyses of TBL protein family in rose with *Arabidopsis thaliana* TBL protein family. Genes marked with pink have been shown to be involved in *O*-acetylation of cell wall polysaccharides in *A. thaliana* (Table 3); genes marked with green may be involved in the defense of roses against *B. cinerea* and were knocked down by virus-induced gene silencing (VIGS) in rose petals. Arrows refer to the well-known TBL proteins in Arabidopsis. The bootstrap values are indicated on the nodes of the branches.

A total of 46 TBLs were identified on *Arabidopsis thaliana* and many of them have been established as *O*-acetyltransferases or potential *O*-acetyltransferase genes (Table 3). To evaluate the relationship between the TBL proteins of rose and *A. thaliana*, a compound phylogenetic tree was constructed using the full-length protein sequences of 46 Arabidopsis and 50 rose TBLs by the ML method. We found that most of the Arabidopsis TBL proteins had at least one rose homologue. *AtTBL* members identified as affecting the *O*-acetylation of xyloglucan, xylan, mannan, and pectin, respectively, were clustered in different branches, suggesting the correctness of our evolutionary tree (Figure 3).

**Table 3:** Arabidopsis TBL family genes involved in acetylation.

Gene name	Gene ID	Polysaccharides	References
AtTBL3	At5G01360	Xylan	Yuan et al., 2016c
AtTBL28	At2G40150	Xylan	Zhong et al., 2017a
AtTBL29/ESK1	At3G55990	Xylan	Xiong et al., 2013
AtTBL30	At2G40160	Xylan	Zhong et al., 2017a
AtTBL31	At1G73140	Xylan	Yuan et al., 2016c
AtTBL32	At3G11030	Xylan	Yuan et al., 2016a
AtTBL33	At2G40320	Xylan	Yuan et al., 2016a
AtTBL34	At2G38320	Xylan	Yuan et al., 2016b
AtTBL35	At5G01620	Xylan	Yuan et al., 2016b
AtTBL19	AT5G15900	Xyloglucan	Zhong et al., 2020
AtTBL20	AT3G02440	Xyloglucan	Zhong et al., 2020
AtTBL21	AT5G15890	Xyloglucan	Zhong et al., 2020
AtTBL22/AXY4L	At3G28150	Xyloglucan	Gille et al., 2011a
AtTBL27/AXY4	At1G70230	Xyloglucan	Gille et al., 2011a
AtTBL23	At4G11090	Mannan	Zhong et al., 2018b
AtTBL24	At4G23790	Mannan	Zhong et al., 2018b
AtTBL25	At1G01430	Mannan	Zhong et al., 2018b
AtTBL26	At4G01080	Mannan	Zhong et al., 2018b
AtTBL10	At3G06080	Rhamnogalacturonan-I	Stranne et al., 2018b
AtTBL44/PMR5	At5G58600	Homogalacturonan	Chiniquy et al., 2019b
TBR/TBL46	TBR/TBL46	Pectin	Bischoff et al., 2010a

**Figure 4:**
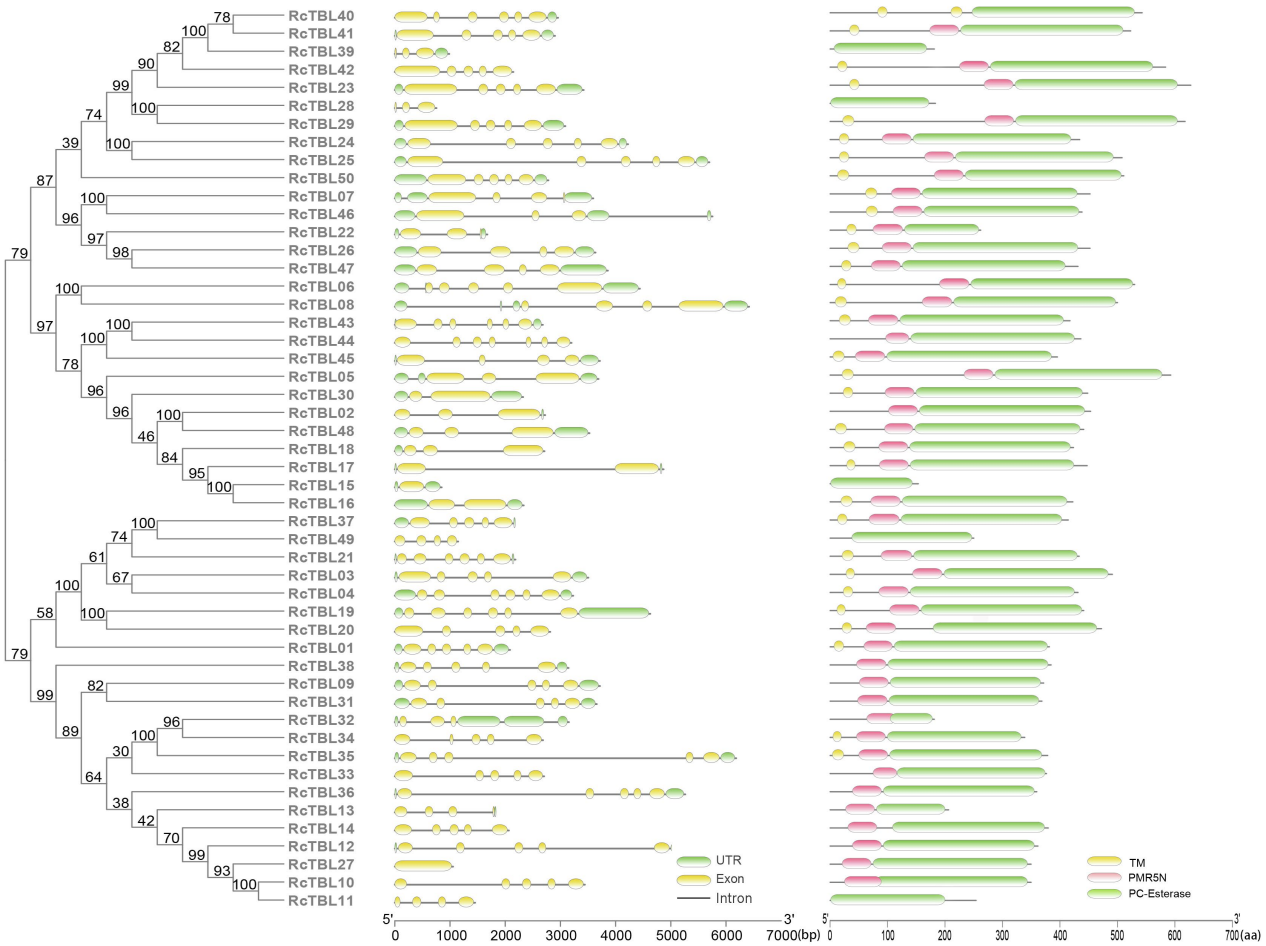
Phylogenetic analyses and gene structures of rose TBL proteins. Complete alignments of all rose TBL proteins were used to construct a phylogenetic tree using the maximum likelihood method. The bootstrap values are indicated on the nodes of the branches. Exon–intron structure of *RcTBLs* is shown in the middle part of the figure, and the green boxes, yellow boxes, and gray lines represent UTRs, exons, and introns, respectively. Transmembrane and conserved domain of *RcTBLs* are displayed in the right part of the figure, and the green boxes, yellow boxes, and pink boxes represent PC-Esterase domain, transmembrane domain, and PMR5N domain, respectively. The scale on the bottom is provided as a reference.

Analysis of the exon–intron structure revealed that the intron structure of *RcTBL*s were highly variable, ranging from 1 to 6, with the largest number (23) of *RcTBL*s containing four introns. In addition, the length of *RcTBL* introns was extremely varied, ranging from tens to thousands of nucleotides. *RcTBL35* contained the longest intron (4,191bp), while the shortest intron (69bp) was present on *RcTBL36* (Figure 4).

The protein sequences of RcTBLs were examined with Pfam and all 50 candidate genes had the PC-esterase domain. Surprisingly, 88% of RcTBLs possessed a cys-rich domain called the PMR5N domain in Pfam (PF14416) that preceded the PC-esterase domain, implying that it could be a crucial structural element of the TBL family. Furthermore, 64% of all 50 RcTBL candidates had at least one transmembrane domain, with RcTBL40 having two transmembrane domains, and 18 RCTBLs had no transmembrane domain (Figure 4).

### The Expression of *RcTBL* Genes in Response to *B. cinerea* Infection

**Figure 5:**
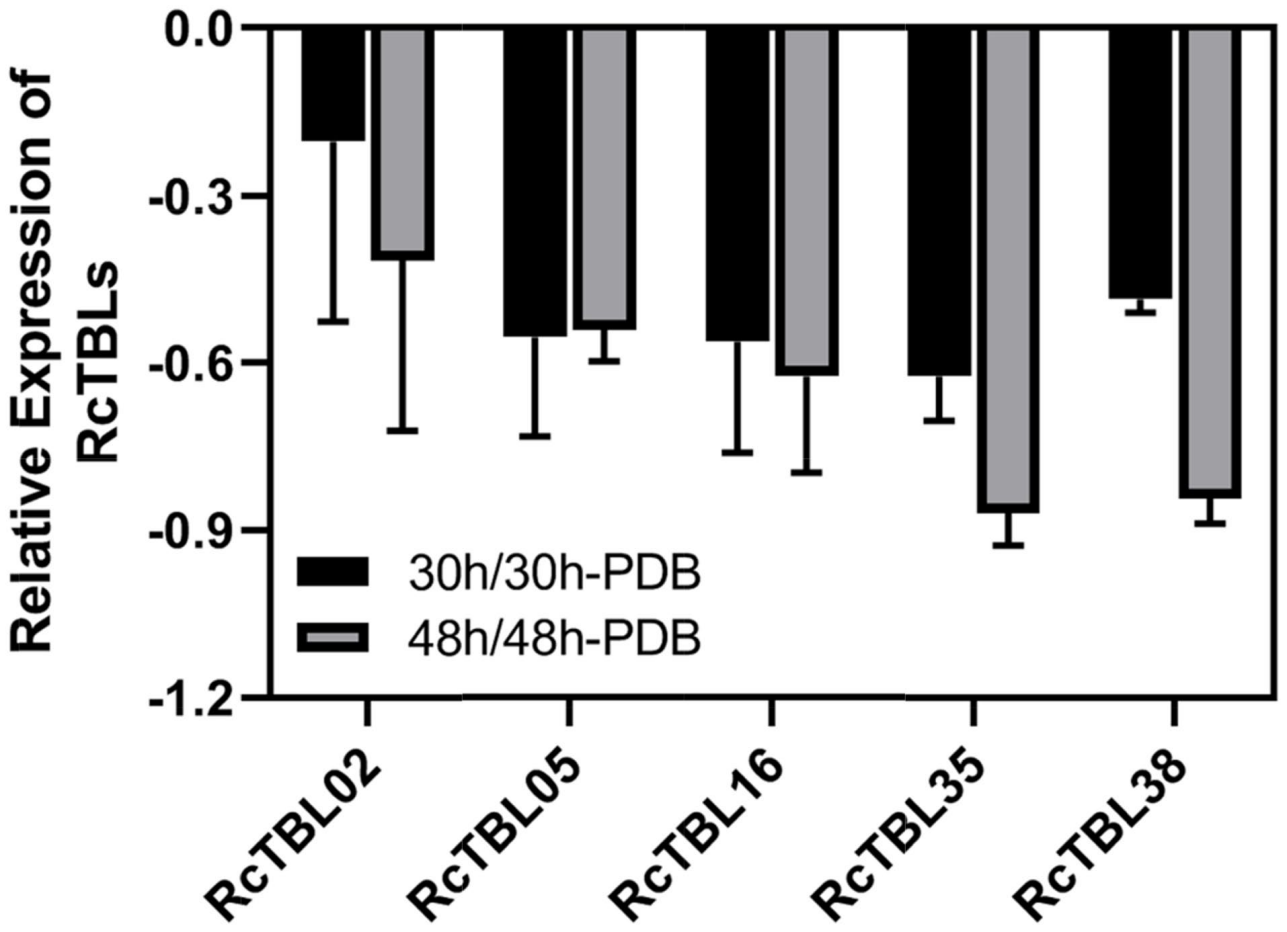
Validation of RNA-Seq results using RT-qPCR. *RcUBI2* was used as an internal control. Expression profile data of five *RcTBL* genes at 30hpi and 48hpi after *B. cinerea* inoculation were obtained using RT-qPCR. Values are the means of three technical replicates ± SD. The used primers are listed in Supplementary Table S1.

Growing evidence has shown that TBL plays an important role in pathogen defense. In order to investigate the role of *RcTBL*s in *B. cinerea* resistance, we obtained RNAseq transcriptomics data from rose petals at 30hpi (hours post-inoculation) and 48hpi (Liu et al., 2018). The 30hpi represents the early response to the infection of *B. cinerea*, while the 48hpi corresponds to the late response. A total of 13 *RcTBL* genes showed significant changes in expression and, interestingly, they were mainly down-regulated in expression (Table 4). Among them, *RcTBL12* and *RcTBL35* were both considerably down-regulated at 30hpi and 48hpi, whereas *RcTBL02*, *RcTBL04*, *RcTBL05*, *RcTBL16*, and *RcTBL36* were only significantly down-regulated at 30hpi, *RcTBL23*, *RcTBL38*, and *RcTBL48* were only significantly down-regulated at 48hpi. Surprisingly, *RcTBL18* expression was dramatically decreased at 30hpi but significantly increased at 48hpi, whereas *RcTBL06* and *RcTBL09* expression was greatly increased at 48hpi. These genes, which are strongly activated by gray mold, could be crucial in rose resistance to *B. cinerea* infection. To further validate the RNA-seq expression profile, the expressions of five *RcTBL*s were examined using RT-qPCR. The RT-qPCR analysis results were found associated with the transcriptome analysis expression patterns (Figure 5).

**Table 4:** Expression patterns of *RcTBL* genes under infection of *B. cinerea*^1^.

Gene^2^	Accession number	Log^2^ratio 30hpi	Log^2^ratio 48hpi^3^
**RcTBL02**	RchiOBHm_Chr1g0359011	−1.450	–
RcTBL04	RchiOBHm_Chr1g0371061	−1.14643	0.405416
RcTBL05	RchiOBHm_Chr1g0375151	−1.210	−0.707
RcTBL06	RchiOBHm_Chr2g0095401	−0.366	1.237
**RcTBL09**	RchiOBHm_Chr2g0129071	0.726	1.139
RcTBL12	RchiOBHm_Chr2g0131441	−1.646	−1.875
RcTBL16	RchiOBHm_Chr2g0163771	−2.239	–
**RcTBL18**	RchiOBHm_Chr2g0170621	−1.639	1.416
RcTBL23	RchiOBHm_Chr3g0479331	−0.682	−1.012
RcTBL35	RchiOBHm_Chr5g0029601	−2.017	−1.643
RcTBL36	RchiOBHm_Chr5g0029611	−1.079	–
RcTBL38	RchiOBHm_Chr5g0070191	−0.505	−2.356
**RcTBL48**	RchiOBHm_Chr7g0195391	−0.361	−1.129

1 *The log2 transformed expression profiles were obtained from the RNA-seq dataset (Liu et al., 2018)*.

2 *The RcTBLs undergo duplicate events are marked in bold*.

3 *A dash ‘-’ means that data are not available*.

### *RcTBL16* Involving in the Defense of Rose Against *B. cinerea*

**Figure 6:**
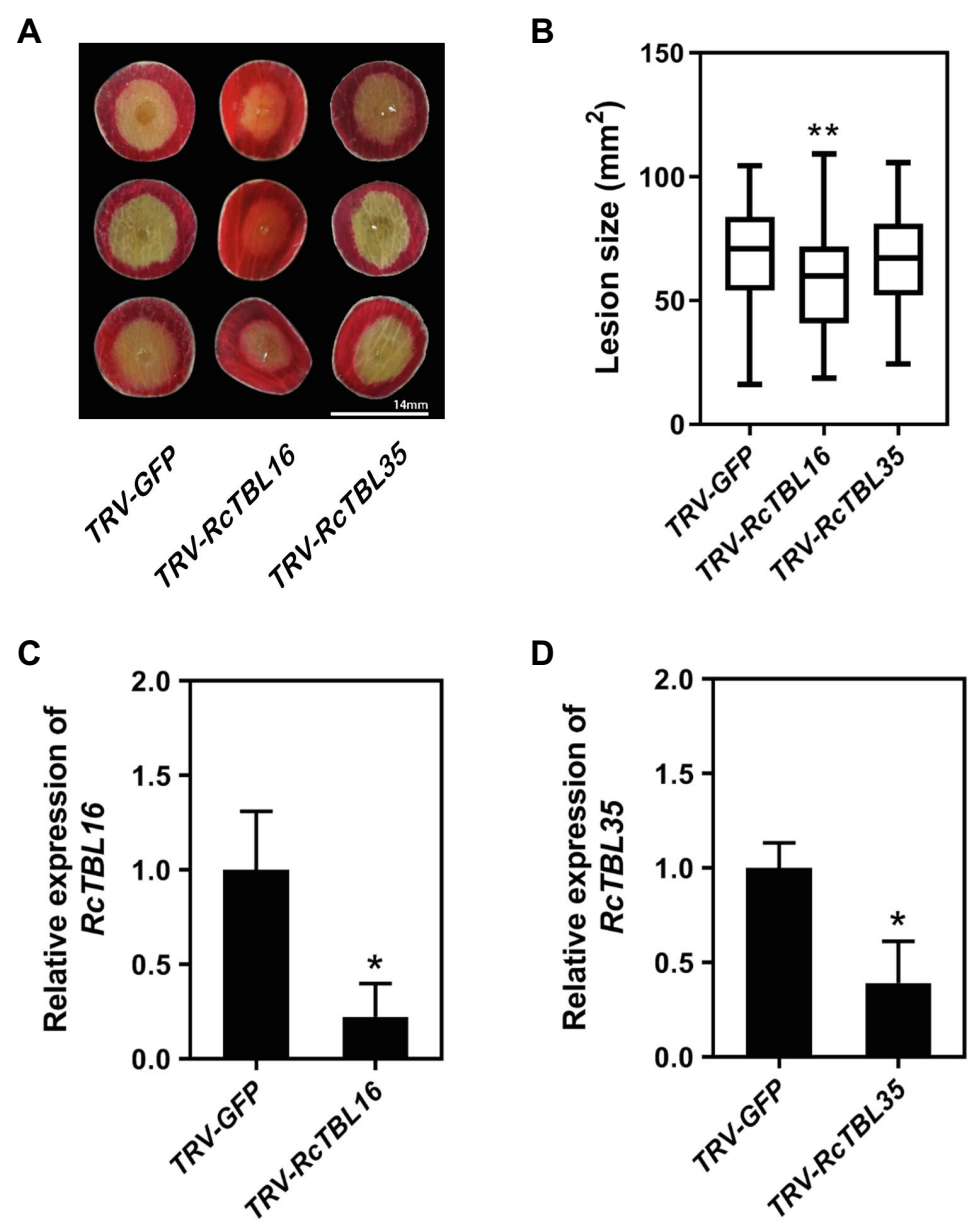
Functional analysis of *RcTBL16*. **(A)** Altered *B. cinerea* resistance upon silencing of *RcTBL16* and unaltered *B. cinerea* resistance upon silenced *RcTBL35* at 60hpi post-inoculation. **(B)** Quantification of *B. cinerea* disease lesions on *TRV-RcTBL16*, *TRV-RcTBL35*, and *TRV-GFP*-inoculated rose petal discs. The graph shows the lesion size from one replicate out of four (*n*≥48) with the standard deviation. **(C)** Quantification of *RcTBL16* expression in *TRV-RcTBL16* and *TRV-GFP*-inoculated petal discs. **(D)** Quantification of *RcTBL35* expression in *TRV-RcTBL35* and *TRV- GFP*-inoculated petal discs. Statistical analyses were performed using Student’s *t* test; ^*^p<0.01, ^*^p<0.05.

The elucidation of gene expression patterns can provide important clues about gene function. Based on the expression results in response to *B. cinerea*, 13 *RcTBL*s with significant differential expression were considered as potential candidates participating in rose against *B. cinerea*. Their potential role in resistance to this fungus was further illustrated by the reduced expression in rose petals by VIGS. We selected *RcTBL16* and *RcTBL35* as candidate genes to explore the relationship between TBL family and rose resistance to *B. cinerea*, because (1) apart from *RcTBL38* (a *PMR5* homolog), *RcTBL16* and *RcTBL35* were the two of maximum down-regulated expressed *RcTBL* genes in response to *B. cinerea* (Table 4), and the results of RT-qPCR support this result (Figure 5); (2) both *RcTBL16* and *RcTBL35* are down-regulated in expression at 30hpi, which represent an early stages of *B. cinerea* infection (Liu et al., 2018).
In order to silence *RcTBL16* and *RcTBL35*, we cloned approximately 200bp fragments of them into the *pTRV2* vector to generate *pTRV2-RcTBL16* and *pTRV2-RcTBL35*. Next, *Agrobacterium tumefaciens* carrying *pTRV2-RcTBL16*, *pTRV2-RcTBL35* and *pTRV1* were co-infiltrated into the rose petals to generate silent rose petals. Then, the infiltrated rose petal discs were put on agar medium and inoculated with *B. cinerea*. Comparing the control inoculated with *TRV-GFP*, petals inoculated with *TRV-RcTBL16* showed notably reduced disease symptoms, whereas the area of disease spots on petals inoculated with *TRV-RcTBL35* had no significantly changes (Figure 6). This result indicated that *RcTBL16* is a susceptibility gene involved in *Botrytis*-rose interaction.

## Discussion

*O*-acetylation is a common modification on plant cell walls and is essential for the stability of the polysaccharide network, with a small amount of acetylation modification affecting plant growth and susceptibility to pathogens. TBL is the largest protein family involved in *O*-acetylation in plants. There are 46 TBLs in Arabidopsis (Bischoff et al., 2010a), 18 of which have been identified as *O*-acetyltransferases, involved in the *O*-acetylation of xylan xyloglucan mannan and pectin respectively (Table 3). Several TBL proteins from rice and poplar have also been shown to be xylan *O*-acetyltransferases (Zhong et al., 2018a,d). However, there is still a gap in the comprehensive analysis of the rose TBL gene family, and their function remains to be discovered. Rose genome sequencing project was completed in 2018, enabling genome-wide analysis of the *RcTBL* gene family. In this study, we have comprehensively analyzed the *RcTBL* family, including phylogeny, gene structure, chromosomal location, gene duplication events, sequence characterization, and analysis of expression profiles.

We identified 50 rose TBLs, more than Arabidopsis (46) (Bischoff et al., 2010a) but less than poplar (64) (Zhong et al., 2018d), rice (66) (Gao et al., 2017), tomatoes (69) (Zhong et al., 2020), which indicates that TBL protein family has expanded to varying degrees in different plants during the evolution. In our structural analysis, we found that in addition to the PC-esterase domain, RcTBLs have a cys-rich domain, namely PMR5N domain, which may be another vital characteristic of the TBL family. Gene duplication plays a very important role in the expansion of gene families. Checking the phylogenetic relationships of TBL between rose and Arabidopsis showed that most evolved branches contained different amounts of AtTBL and RcTBL proteins, suggesting that the two species displayed conserved evolution.

The composition of the cell wall is closely correlated with fungal disease resistance, and altered levels of cell wall polysaccharide *O*-acetylation modification can lead to altered plant resistance to fungi. In some cases, a reduction in the level of acetylation enhances plant resistance to pathogens. For instance, Arabidopsis *rwa2* showed a 20% reduction in the degree of cell wall polysaccharide acetylation modification and was more resistant to *B. cinerea* than wild type. In this study, we also found *RcTBL16* negatively regulating resistance to gray mold in rose. *PMR5*, a member of the TBL family, has the same performance in resisting powdery mildew in Arabidopsis (Vogel et al., 2004a), by the *O*-acetylation modification of homogalacturonan (Chiniquy et al., 2019a). Therefore, we consider that partial rose TBL genes may be involved in the susceptibility of rose to *B. cinerea* through acetylating cell wall.

It is clear that changes in the level of *O*-acetylation modification of cell walls can affect plant resistance or susceptibility to pathogens, but the exact mechanism remains a mystery at present. One hypothesis suggests that alterations in the polysaccharide composition of the cell wall will modify the cell wall integrity (CWI) system, thereby triggering plant defenses and activating specific defense responses (Bacete et al., 2017). Alteration of cell wall xylan acetylation caused by *ESK1* impairment was accompanied by an enhanced accumulation of abscisic acid, the constitutive expression of genes encoding antibiotic peptides and enzymes involved in the biosynthesis of tryptophan-derived metabolites, and the accumulation of disease resistance-related secondary metabolites and different osmolites, implying that the damage to CWI triggers defense response of plant (Lugan et al., 2010; Xin et al., 2010; Xu et al., 2014; Escudero et al., 2017). However, it needs to be substantiated by stronger evidence. Overall, more research remains to be done on the certain mechanisms of TBL participant plant–pathogen interactions, but there is no doubt that TBLs in rose is most possibly as a susceptibility factor for the resistance to gray mold, and this result will also provide clues for rose breeding application (i.e., enhancing persistent plant resistance by silencing or knocking out susceptibility genes).

## Conclusion

The study performed a genome-wide analysis of *RcTBL*s, including phylogenetic relationships, collinearity, and expression analysis. A total of 50 non-redundant *RcTBL* members were identified. The expression analysis showed that transcription regulation of several *RcTBL* was reduced by *B. cinerea* infection in rose petals. Based on these analyses and VIGS, we further demonstrated that *RcTBL16* was engaged in susceptibility of rose to gray mold. The information provided by these results can promote the further functional analysis of *RcTBL* genes and application in rose disease resistance breeding.

## Data Availability Statement

Publicly available datasets were analyzed in this study. These data can be found at: https://lipm-browsers.toulouse.inra.fr/pub/RchiOBHm-V2/.

## Author Contributions

ZZ and YT conceived and designed the research and wrote the paper. YT performed the experiments. YT, SZ, XL, and ZZ analyzed the data. All the authors have read and approved the final version of the manuscript. All authors contributed to the article and approved the submitted version.

## Funding

his study was supported by the National Natural Science Foundation of China (grant number 31772344) to ZZ. It is further supported by the Science and Technology Program of Beijing Municipality (grant number Z181100002518002). The funders played no role in study design, data collection and analysis, decision to publish, or preparation of the manuscript.

## Conflict of Interest

The authors declare that the research was conducted in the absence of any commercial or financial relationships that could be construed as a potential conflict of interest.

## Publisher’s Note

All claims expressed in this article are solely those of the authors and do not necessarily represent those of their affiliated organizations, or those of the publisher, the editors and the reviewers. Any product that may be evaluated in this article, or claim that may be made by its manufacturer, is not guaranteed or endorsed by the publisher.

## Footnotes

^1^ http://pfam.xfam.org

^2^ https://lipm-browsers.toulouse.inra.fr/pub/RchiOBHm-V2/

## Abbreviations

hpi: hours post inoculation
ML: maximum likelihood
RPKM: number of reads per kb per million reads
HMM: hidden Markov model
CWI: cell wall integrity
aa: amino acids
VIGS: virus-induced gene silencing
