# Supplementary material for: Global Investigation of TBL Gene Family in Rose (Rosa chinensis) Unveils RcTBL16 Is a Susceptibility Gene in Gray Mold Resistance
Source: Front Plant Sci. 2021 Oct 1;12:738880. doi: 10.3389/fpls.2021.738880 (PMC8575163; doi:10.3389/fpls.2021.738880)
Supplement: Supplementary file 1 [file Table_1.docx]

**Supplemental Table S1** List of primers used in this study

| **Gene name** | **Purpose** | **Primer sequence（5’-3’）** |  |
| --- | --- | --- | --- |
| RcTBL16-VIGS | VIGS | F:GGGGACAAGTTTGTACAAAAAAGCAGGCTGCTCAAAGCAGCACAAAACCA |  |
|  |  | R:GGGGACCACTTTGTACAAGAAAGCTGGGTTGCAGCAAAATCTCATTCCATG |  |
| RcTBL35-VIGS | VIGS | F:GGGGACAAGTTTGTACAAAAAAGCAGGCTGTCCCTGATACCTGGAATGAAC |  |
|  |  | R:GGGGACCACTTTGTACAAGAAAGCTGGGTGGATGCTTACTCTTCCTTGATG |  |
| RcTBL02 | RT-qPCR | F:AATGCTCTCCGCCTGGTTTT |  |
|  |  | R:CTCAAAGTGGTCAGGCGTCG |  |
| RcTBL05 | RT-qPCR | F:CGATGCTCACTGCTCTTGCTAC |  |
|  |  | R:CCCCGCCTTCATAATGGTC |  |
| RcTBL16 | RT-qPCR | F:TCTATGGGCTCCTTACTTGGTTA |  |
|  |  | R:GAATCTCTGCCGCATACTTACCCCA |  |
| RcTBL35 | RT-qPCR | F:GTTCTCCTAGCATTGTGCCTTCT |  |
|  |  | R:GCGACTCATTCACCTGCTTCT |  |
| RcTBL38 | RT-qPCR | F:ATGGATCGCTTGGCTGCTT |  |
|  |  | R:AAAACTCGGGTCCTTGTGGTAT |  |
| RcUBI2 | RT-qPCR | F:GCCCTGGTGCGTTCCCAACTG |  |
|  |  | R:CCTGCGTGTCTGTCCGCATTG |  |
